# Is there plasticity in developmental instability? The effect of daily thermal fluctuations in an ectotherm

**DOI:** 10.1002/ece3.3556

**Published:** 2017-11-02

**Authors:** Øystein Nordeide Kielland, Claus Bech, Sigurd Einum

**Affiliations:** ^1^ Department of Biology Centre for Biodiversity Dynamics Norwegian University of Science and Technology, NTNU Trondheim Norway; ^2^ Department of Biology Norwegian University of Science and Technology, NTNU Trondheim Norway

**Keywords:** allometry, bioenergetics, climate change, development, heritability, ontogeny, quantitative genetics

## Abstract

Diversified bet‐hedging (DBH) by production of within‐genotype phenotypic variance may evolve to maximize fitness in stochastic environments. Bet‐hedging is generally associated with parental effects, but phenotypic variation may also develop throughout life via developmental instability (DI). This opens for the possibility of a within‐generation mechanism creating DBH during the lifetime of individuals. If so, DI could in fact be a plastic trait itself; if a fluctuating environment indicates uncertainty about future conditions, sensing such fluctuations could trigger DI as a DBH response. However, this possibility has received little empirical attention. Here, we test whether fluctuating environments may elicit such a response in the clonally reproducing crustacean *Daphnia magna*. Specifically, we exposed genetically identical individuals to two environments of different thermal stability (stable vs. pronounced daily realistic temperature fluctuations) and tested for effects on DI in body mass and metabolic rate shortly before maturation. Furthermore, we also estimated the genetic variation in DI. Interestingly, fluctuating temperatures did not affect body mass, but metabolic rate decreased. We found no evidence for plasticity in DI in response to environmental fluctuations. The lack of plasticity was common to all genotypes, and for both traits studied. However, we found considerable evolvability for DI, which implies a general evolutionary potential for DBH under selection for increased phenotypic variance.

## 1 INTRODUCTION

When the environment changes throughout the lifetime of an organism, there is an increased potential for a mismatch between the expressed and optimal phenotype. If the changes are predictable and infrequent (such as seasonal changes; e.g., onset of winter), reversible phenotypic plasticity may represent an option to track the environment, by always changing to express the optimal phenotype (DeWitt, Sih, & Wilson, 1998). Phenotypic plasticity may also mediate adaptive changes on a shorter temporal scale (such as daily fluctuations in light or temperature) if the costs involved do not exceed the fitness benefits. However, if environmental changes occur too frequently, too unpredictably, or by too large a magnitude (Bozinovic, Medina, Alruiz, Cavieres, & Sabat, 2016; Dowd, King, & Denny, 2015; Kern, Cramp, & Franklin, 2015), costs of phenotypic plasticity open for adaptive alternatives of higher evolutionary value (Botero, Weissing, Wright, & Rubenstein, 2015).

Bet‐hedging represents one such alternative biological mechanism that organisms may evolve to maximize long‐term (geometric) mean fitness in stochastic environments (Slatkin, 1974; Starrfelt & Kokko, 2012). Specifically, diversified bet‐hedging (DBH), whereby a single mother produces a range of offspring phenotypes, may be advantageous if it ensures that some of these are well adapted under any environmental conditions (Einum & Fleming, 2004; Kaplan & Cooper, 1984; Starrfelt & Kokko, 2012). An example of this is found in planktonic rotifers that live in temporary ponds. Rotifer populations survive dry periods as dormant resting eggs, which hatch as water levels return to normal levels. However, to ensure long‐term survival of a genotype in an unpredictable environment where the duration of the water covered period is sometimes too short (<10 days) to allow for resting egg production, not all eggs should hatch after the first dormancy period. Thus, a DBH response to such conditions should be to decrease hatching rates, whereas under predictable environments, hatching rates should be high. Such evolutionary responses have been demonstrated experimentally (Tarazona, García‐Roger, & Carmona, 2017). Bet‐hedging is generally associated with parental effects, and a common observation is that the parental influence on offspring phenotypes declines through ontogeny of the offspring (Einum & Fleming, 2000; Lindholm, Hunt, & Brooks, 2006; Wilson & Réale, 2006). Instrumental in defining phenotypic variation, among individuals of a single genotype, is that variation may also develop throughout the life of organisms. This may occur even if they experience equal environmental conditions. In quantitative genetics, such phenotypic variation within genotypes is often referred to as developmental instability (DI; Graham, Emlen, & Freeman, 1993; Falconer & Mackay, 1996). DI can have a genetic (Lynch & Gabriel, 1987; Pélabon, Hansen, Carter, & Houle, 2010), micro‐environmental (Lajus, Graham, & Kozhara, 2003), or intrinsic stochastic developmental source (Hansen, Carter, & Pélabon, 2006; Lajus et al., 2003).

It has been suggested that DI has evolved as a bet‐hedging mechanism to maximize long‐term fitness in a fluctuating or heterogeneous environment (Botero et al., 2015; Scheiner, 2014a; Simons & Johnston, 1997; Tufto, 2015). This opens up for the possibility of a within‐generation mechanism creating DBH during the lifetime of individuals, rather than being determined by parental effects (Lajus et al., 2003; Scheiner, 2014b). If so, DI could in fact also have a plastic component; if a fluctuating environment indicates uncertainty about future conditions, sensing such fluctuations could trigger DI as a within‐generational DBH response. Whether or not short timescale fluctuating environments can function as a stochastic cue remains unknown, and this possibility has received little, if any, empirical attention. It may be argued that environmental influence on DI has been studied within the field of fluctuating or directional asymmetry (FA, DA; e.g., Polak, 1993; Hendrickx, Maelfait, & Lens, 2003; Moller, 2006), a commonly used measure of DI. These two measures of asymmetry are general descriptions of the degree of asymmetrical development in a bilateral character (Van Valen, 1962). However, whereas the increase in DI under environmental stochasticity can be hypothesized to represent an adaptive DBH response, an increase in asymmetry is unlikely to be adaptive (Moller, 1997; Pelabon, Carlson, Hansen, Yoccoz, & Armbruster, 2004; Pelabon & Hansen, 2008; Wagner, Booth, & Bagheri, 1997). Hence, studies on how the environment influences within‐genotype variance, using FA or DA, cannot be applied to infer adaptive DBH responses.

Temperature effects in ectotherms provide a malleable system within which this topic can be studied. The performance of a wide range of fitness‐related traits is highly influenced in a direct manner by the environmental temperature ectotherms experience, including responses not only to changes in mean temperature, but also to levels of temperature fluctuations (Brodte, Knust, & Pörtner, 2006; Callaghan, Tunnah, Currie, & MacCormack, 2016; Gillooly, Brown, West, Savage, & Charnov, 2001; Kern et al., 2015; Schaefer & Ryan, 2006). One such trait, growth, can sometimes essentially function as a proxy for fitness (Lampert & Trubetskova, 1996). Growth is dependent on the surplus energy from metabolism (e.g., Angilletta & Dunham, 2003). Thus, to counter negative fitness effects, metabolic adaptations to changes in thermal mean and variability should evolve, which include active acclimation mechanisms (e.g., up‐ and downregulation of metabolic rate (MR) and/or production of heat‐shock proteins; Feder & Hofmann, 1999; Johnston & Dunn, 1987; Kielland, Bech, & Einum, 2017; White, Alton, & Frappell, 2012). However, it is not known whether temperature variability influences levels of phenotypic variation within genotypes.

**Figure 1:**
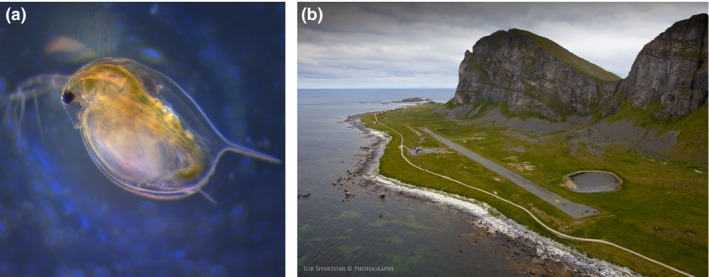
(a) The model organism, *Daphnia magna*, is a small planktonic crustacean that reproduces by alternating between cyclical parthenogenesis and sexual reproduction. The asexual reproduction generally continues indefinitely under favorable conditions, while the sexual reproductive bouts occur when the environment becomes unfavorable. Photograph credits: Ø.N. Kielland (b) The study site origin at Værøy (Sandtjønna, to the right), as seen from the air. Photograph credits: Tor Sivertstøl, www.lofotor.com. Photo permission is valid for one single publication, web only

In this study, we test whether the level of environmental fluctuations experienced influences DI, which would be a prerequisite for DBH to operate within generations. Using a clonal model organism (*Daphnia magna*, Figure 1a), we are able to obtain within‐genotype levels of phenotypic variation in two fitness‐related and temperature‐dependent traits (somatic body mass and metabolic rate) under contrasting environmental regimes (stable vs. fluctuating temperatures).

## 2 METHODS

### 2.1 Animals

The study population originated from Sandtjønna (67°41′12.8″N 12°40′19.2″E, Figure 1b), which is a small, shallow (maximum depth <1 m) pond on the Værøy Island, northern Norway. Ephippia containing resting eggs, resulting from sexual reproduction of *D. magna,* were collected from Sandtjønna in November 2014. Twenty such ephippia were hatched in the laboratory and hatchlings propagated by asexual reproduction. The resulting isofemale populations formed the basis of 20 genotypes, hereafter referred to as clones. Stock animals were kept in 2.5‐L aquaria containing a selenium dioxide altered version of ADaM (Aachen Daphnia Medium; Kluttgen, Dulmer, Engels, & Ratte, 1994), in a 17°C climate room at Norwegian University of Science and Technology, Trondheim, Norway. The photoperiod followed a 16 light (L): 8 dark (D) cycle, and animals were kept in these conditions through multiple asexual generations (generation time: ~14–18 days) for a year before the experiment started. Medium was exchanged weekly, and animals were fed three times a week with Shellfish Diet 1800^®^ (Reed mariculture Inc.) at a final concentration in the aquaria of 2.4 × 10^5^ cells/ml.

### 2.2 Protocol

**Figure 2:**
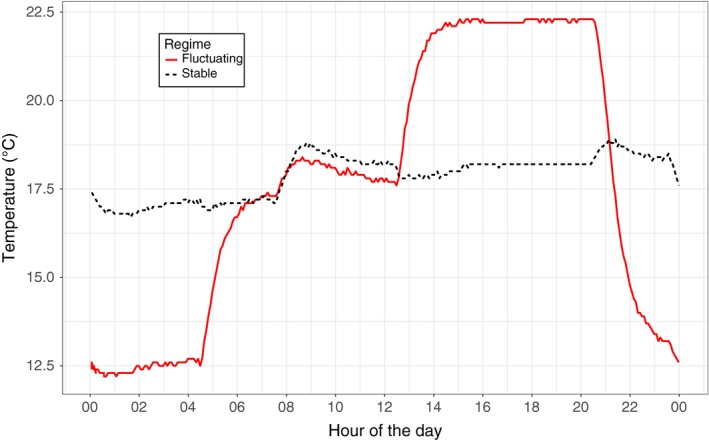
Temperature log data from the climate cabinets. Shown above is the daily variation within the stable (black, dashed line) and fluctuating (red, solid line) temperature treatments. Aquatic temperature deviated slightly from the ambient temperature, where the mean temperature was 17.8°C in both treatments. The light period started at 08.00 and ended at 00.00. This probably explains some of the observed temperature pattern, as the temperatures were logged in a 50‐ml plastic centrifuge tube

Three offspring from a single newborn clutch (<36 hr old) were randomly selected from each of the 20 clones. These were then assigned to one of three treatments. In the first treatment, metabolic rate (MR) and body mass (BM) were measured at 17°C immediately after assignment. This measure provides a baseline level of phenotypic variance at birth. The two remaining individuals were allowed to grow for 5 days (until shortly before maturation) prior to measurements of MR and BM in either (1) a stable thermal environment (mean aquatic temperature 17.8 ± 0.6°C) or (2) a fluctuating thermal environment (mean ± *SD* aquatic temperature 17.8 ± 3.8°C). The latter environment was obtained by keeping the air temperatures at 17°C from 05.00 to 13.00, at 22°C from 13.00 to 21.00 and at 12°C from 21.00 to 05.00 (see Figure 2). Hence, the mean temperatures in the fluctuating and stable treatments were equal. The experienced levels of variation in the fluctuating treatment were within realistic daily thermal fluctuation ranges, as observed in their native environment (see Appendix S1 for details). The rationale for using predictable daily thermal fluctuations was that for water bodies such fluctuations indicate the sensitivity of water temperatures to weather conditions, and hence, high daily fluctuations would represent an environment highly sensitive to stochastic weather changes. Individuals were kept separately in 50‐ml plastic centrifuge tubes (VWR International, USA) under 16L:8D light regimes and were fed ad libitum at a final concentration of 2 × 10^5^ cells/ml on day 0 (first day; at birth), day 2, and day 4. The experiment was repeated in 12 blocks, and on average, 83% of the clones were present in each of these. One of the 12 experimental blocks showed abnormally low growth for all individuals and was thus excluded from the data set (*n* = 51). In addition, five animals that failed to grow during the 5 days of the experiment were excluded.

### 2.3 Respiration and body mass measurements

All metabolic rate measurements were made at 17°C in a dark climate cabinet. During the measurements, daphnids were kept individually in ~200‐μl glass chambers (Loligo^®^ systems, Denmark) that were sealed using adhesive PCR‐film (Thermo Scientific Inc., USA). The decline in oxygen content was then measured optically of up to 20 individuals simultaneously using pO_2_‐dependent fluorescence technology (SDR SensorDish© Reader, PreSens GmbH, Germany). Respiration of newborns was measured for 3 hr, while the larger (day 5) animals were measured for 1.5 hr. The lengths of the animals (GL; gut length, measured from the apex of the foregut to the base of the hindgut) were measured to the nearest 0.01 mm using photographs from a stereo microscope (Leica Microsystems GmbH, Germany) and the software ImageJ (Rasband, 1997‐2016). Body masses (BM, dry weight, mg) were estimated by linear regression, using previously measured data on gut length (GL) and BM (Yashchenko, Fossen, Kielland, & Einum, 2016): BM = 0.00681 × GL^2.75^ (*df* = 30, *r* ^2^ = 0.99, *p* < .001). Details regarding the respirometric and the BM‐GL regression procedure are given by Yashchenko et al. (2016).

### 2.4 Genetic variance

In the current experiment, we used the broad‐sense version of evolvability (mean scaled *V*_*G*_; genetic variance (Houle, 1992; Hansen, Pélabon, & Houle, 2011)) to approximately illustrate the quantity of genetic variation for our given population. For that reason, we might overestimate the evolutionary potential, as overall broad‐sense evolvabilities might be higher than the narrow‐sense evolvability, which exclusively considers the additive genetic variance. Thus, the evolvability estimates should be viewed as rough quantitative estimates on the genetic variance. For clonal organisms, the broad‐sense evolvability is obtained in a linear mixed model, by having genotype as a random effect (*n* = 20 groups) and log‐transforming the response variable. Evolvability (in the narrow‐sense, mean scaled *V*_*A*_; additive variance) represents expected proportional change in population mean trait, for a unit strength (mean‐standardized) directional selection (Hansen, Pélabon, Armbruster, & Carlson, 2003; Hereford, Hansen, & Houle, 2004; Matsumura, Arlinghaus, & Dieckmann, 2012). Measures of evolvability are convenient for doing comparative analyses on evolutionary potential, as any trait's mean, μ, can be predicted to change by a factor (1 + *e*_μ_β_μ_)^*t*^ over *t* generations, where *e* is the evolvability and β is the strength of selection on the mean trait value μ (Hansen, 2013).

### 2.5 Statistics

The data were analyzed in a linear mixed effect model (LME) using the statistical software *R* (R Core Team, 2017) and the package *nlme (Pinheiro, Bates, DebRoy, & Sarkar,* 2017). This was used to obtain estimates on broad‐sense evolvability (genetic variance) in BM and MR and to control for random run effects. Within each of the 11 experimental blocks, MR was measured in three different runs: one at birth and the remaining two runs at day 5. The two runs on day 5 were due to logistical reasons, where MR of up to 20 individuals could be measured simultaneously (see *Respiration and body mass measurements*). Thus, run number was included as a random factor (for a total of 11 × 3 = 33 runs), incorporating both variation among blocks and among runs within blocks. Due to the variation in clone representation across runs, clone was modeled as nested within run. The full models are given by:

(1) $$\begin{array}{cc} \text{log}{\text{MR}}_{ijkl} & =\beta_{1}\times {\text{Treatment}}_{l}+\beta_{2}\times \text{log}{\text{BM}}_{ijk}+\beta_{3}\times {\text{Treatment}}_{l}\;:\;\text{log}{\text{BM}}_{ijk}\\ & +\alpha_{k}+\alpha_{j|k}+\varepsilon_{ijkl} \end{array}$$

(2) $$\text{log}{\text{BM}}_{ijkl}=\beta_{1}\times {\text{Treatment}}_{l}+\alpha_{k}+\alpha_{j|k}+\varepsilon_{ijkl}$$

where β's are parameter estimates for the fixed effects, α_*k*_ and α_*j*|*k*_ are variance terms for random run effects and clone effects nested within run, respectively*,* and ε corresponds to residuals for individuals *i* of clone *j *=* *1, …., 20 in run *k *=* *1, …., 33 and treatment *l* = 1, 2, 3 (day 0, day 5 stable and day 5 variable). In these models, the variance of the residuals (ε) represents our measure of DI (i.e., variance within clones). This variance of the residuals was allowed to differ both among clones and treatments using the VarIdent command from the nlme package. Specifically, when analyzing the effect of fluctuating temperatures on DI, we used a subset of the data that exclusively contained animals measured at day 5. Here, we also allowed for an interaction between clone and treatment on the weighted variance, where a significant interaction would indicate a clone‐specific response to fluctuating environments. A common fixed effect for both MR and BM was treatment (β_1_; Equations 1 and 2). For MR, the fixed part also included the allometric scaling between body mass and metabolic rate (log MR ~ α + β_2_ × log BM; Equation 1) and its interaction with treatment (β_3_; Equation 1). Model selection followed a backward selection procedure, with significance of first random (including variance of residuals among clones and treatments) and then fixed effects being assessed through likelihood ratio tests (LRT, Zuur, Ieno, Walker, Saveliev, & Smith, 2009). Here, full models were replaced by the nested model that represented the least change in likelihood, where each candidate model was tested separately. The final model in this process had no parameters that could be dropped without causing a significant decrease in likelihood.

## 3 RESULTS

### 3.1 Metabolic rate

**Figure 3:**
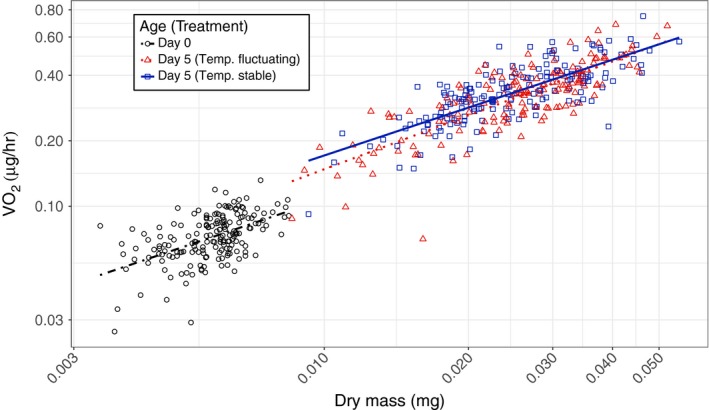
Metabolic rate (VO_2_) of *Daphnia magna* (*n* = 573) at birth (day 0, black circles) and after 5 days of growth (day 5). During these 5 days, animals either experienced a stable temperature regime of 17°C (blue squares) or daily fluctuating temperatures between 12, 17 and 22°C (mean 17°C; red triangles)

**Figure 4:**
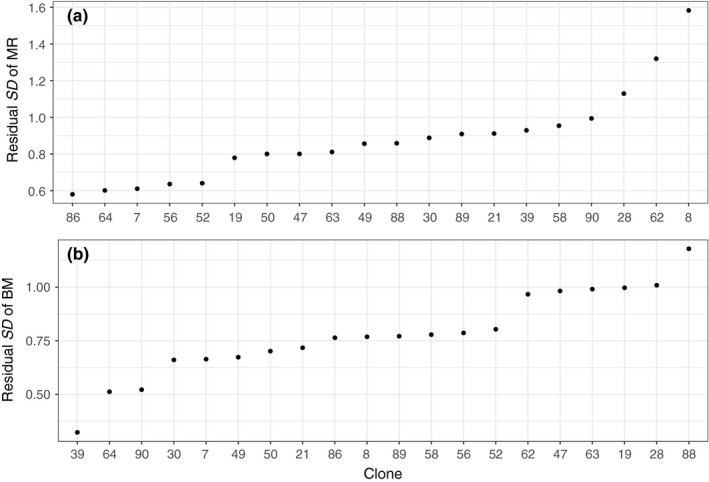
Developmental instability (DI, residual standard deviation) in (a) metabolic rate (MR) and (b) body mass (BM) in *Daphnia magna* (*n* = 336) after 5 days of growth (day 5). The residuals derive from the best models of the two traits, but excluding the term quantifying separate within‐genotype (clone) variance for the different clones

The mean allometric slope describing the relationship between log MR and log BM (β = 0.77, *p* < .001, Figure 3) did not differ between the different temperature treatments (fluctuating vs. stable temperature, *p* = .11) nor between the two age classes (*p* = .22). The metabolic rate intercept was higher at day 5 than at day 0 (*p* < .001). Furthermore, at day 5 there was a significant effect of temperature regime, with animals at fluctuating temperatures showing a lower MR than those from a stable temperature (*p* < .01, Figure 3). Stratifying variance to differ among treatments did not improve the model, hence stable and fluctuating temperatures did not differ significantly in the amount of DI (DI parameters = 2, $\sigma_{\mathrm{stable}}^{2}$ = 0.87 × $\sigma_{\mathrm{fluct}.}^{2}$_,_ *p* = .19). However, clones varied significantly in the amount of DI (DI parameters = 20, $\sigma_{\mathrm{largest}\;\mathrm{clone}\;\mathrm{DI}}^{2}$ = 2.99 × $\sigma_{\mathrm{smallest}\;\mathrm{clone}\;\mathrm{DI}}^{2}$, *p* < .05, Figure 4a (note: Figure 4 shows *SD* in within‐clone residuals from the model where variance is considered equal for all clones)). However, there was no interaction between clone and treatment on DI (*p* = .49). There was a significant amount of genetic variation (*p* < .05) in MR. The broad‐sense evolvability, *E*_μ_, in MR was estimated to 0.09% (using all data). There was also variation in MR among runs (*p* < .01).

### 3.2 Body mass

DI in body mass (BM, log scaled) did not differ statistically between the two temperature treatments on day 5 (DI parameters = 2, $\sigma_{\mathrm{fluct}.\;\mathrm{temp}.}^{2}$ = 0.77 × $\sigma_{\mathrm{stable}\;\mathrm{temp}.}^{2}$_,_ *p* = .09). Genetic variance in BM was significant (broad‐sense evolvability, using all data: *E*_μ_ = 3.5%, *p* < .001), and the clones differed significantly in DI (DI parameters = 20, $\sigma_{\mathrm{largest}\;\mathrm{clone}\;\mathrm{DI}}^{2}$ = 5.65 × $\sigma_{\mathrm{smallest}\;\mathrm{clone}\;\mathrm{DI}}^{2}$, *p* < .05, Figure 4b). There was no interaction between clone and treatment in DI (*p* = .44). Significant run effects in BM were observed (*p* < .001), but there was no difference in mean BM after 5 days of growth between the temperature treatments (mean ± *SD* dry mass; 26.4 ± 0.8 μg at fluctuating temperatures, 25.7 ± 0.6 μg at stable temperature, *p* = .22).

## 4 DISCUSSION

If DI is a plastic trait that responds to environmental fluctuations, this may contribute to a within‐generational diversifying bet‐hedging (DBH) response. Empirically, plasticity in DI would be observable through differences in within‐clone phenotypic variation among environments that differ in their stability. However, we found no such effects, neither in body mass nor in metabolic rate, when *Daphnia* were exposed to different levels of thermal fluctuations. Furthermore, this lack of plasticity in DI appeared to be general, as there was no clone‐specific response to temperature fluctuations, although the sample size for this test was somewhat modest.

Theoretical models on reaction norms predict that, for a given study system, bet‐hedging, plasticity or genetic evolution have evolved depending on predictability of the cue and on the timescale over which the cue operates (Botero et al., 2015; Scheiner, 2014b; Tufto, 2015). In general, temperature shows high autocorrelation within a season in aquatic systems (Appendix S1, Burgess & Marshall, 2011; Shama, 2015; Kielland et al., 2017). It is therefore not unlikely that our study population has evolved to respond to predictable cues, and for that reason, it does not possess the within‐generation plasticity in DI that may act as a DBH response. Future work should focus on plasticity in DI of populations that experience a higher degree of stochastic temperature regimes. However, we did find genetic variance in DI, suggesting that it is a trait that may evolve given selection for increased within‐genotype phenotypic variation. In agreement with our results, multiple studies show heritability in within‐genotype phenotypic variation or demonstrate that evolution of DI is indeed plausible (Ayroles et al., 2015; Breno, Bots, & Van Dongen, 2013; Carter & Houle, 2011; Hansen et al., 2006; Leamy, 1997; Pélabon et al., 2010; Polak & Starmer, 2001). As we used broad‐sense evolvability in the present study, the estimated rate of evolution in BM, MR, and DI is expected to be lower if it is calculated using evolvability measured in the narrow‐sense (i.e., phenotypic variation due to additive genetic variance). If we assume the empirical median evolutionary selection gradient value (β) of 0.48 (mean‐standardized, unbiased selection gradient for univariate traits; Hereford et al., 2004), a narrow‐sense evolvability value of, for example, 0.1% represents an evolutionary potential of ~5% change in trait value over 100 generations, or doubling/halving the trait value in ~1,450 generations (Hansen, 2013).

The daily thermal range used in the fluctuating temperature treatment exceeded 95% of the daily ranges the *Daphnia* experience during the growth season in their native environment (Appendix S1). Thus, the fluctuations were realistic but pronounced. Yet, no negative effects were detected on body size shortly before maturation. We cannot exclude the possibility that such costs could occur later in life through shorter life span and/or reduced fecundity (Manenti, Sørensen, Moghadam, & Loeschcke, 2014). However, juvenile‐specific growth rate has previously been shown to be a good proxy for fitness in *Daphnia* sp. (Arbaciauskas, 2004; Lampert & Trubetskova, 1996). Thus, the *Daphnia* from our study population are seemingly well adapted to an environment of high temperature variance on a fine temporal scale. This is also reflected by the relatively small response of the metabolic rate to temperature fluctuations. Our observed decline in metabolic rate under fluctuating temperature mirrors previous studies (Chen & Stillman, 2012; Chown, Haupt, & Sinclair, 2016; Niehaus, Wilson, Seebacher, & Franklin, 2011). According to theories on metabolic homeostasis (“metabolic cold adaptation”; White et al., 2012; Bruneaux et al., 2014), animals should acclimate through downregulation of MR at high temperatures to counter the passive thermal increase in metabolism (Clarke & Johnston, 1999; Kielland et al., 2017). However, they should also upregulate MR at low temperatures. Thus, acclimation of MR under fluctuating temperatures creates a dilemma. As *Daphnia* that experience a fluctuating temperature downregulate the MR, it appears that they prioritize homeostasis at the high temperature (i.e., avoid excessively high MR). One might speculate that this is related to an asymmetric fitness cost of expressing too low MR at a low temperature (i.e., reduced growth rate) vs. too high MR at a high temperature (i.e., increased risk of mortality due to insufficient oxygen availability).

To conclude, we find no evidence that plasticity in DI, in response to environmental fluctuations, contributes to DBH in *Daphnia*. The lack of plasticity was a general property of the population, and for both traits studied (BM and MR). However, we found genetic variance in DI, which implies a general evolutionary potential for DBH under selection for increased phenotypic variance.

## CONFLICT OF INTEREST

None declared.

## AUTHOR CONTRIBUTIONS

The study was conceived and initiated by SE and ØNK, while the experimental work and initial draft of the manuscript was conducted by ØNK. All authors contributed to the study design, analysis of data, and revisions of the manuscript. All authors approved to submit the final version of the manuscript.
