# Supplementary material for: Is there plasticity in developmental instability? The effect of daily thermal fluctuations in an ectotherm
Source: Ecol Evol. 2017 Nov 2;7(24):10567–74. doi: 10.1002/ece3.3556 (PMC5743494; doi:10.1002/ece3.3556)
Supplement: Supplementary file 1 [file ECE3-7-10567-s001.docx]

# Appendix S1

# Temperature data

*Logging of temperature at the study site*

Sandtjønna is located at the island of Værøy (67°41'12.8"N 12°40'19.2"E) in the Lofoten archipelago, Norway. To get an estimate on the magnitude and autocorrelation in temperatures that are normal to experience in the wild, temperatures at the study site was logged. The temperature was logged from April 15^th^ – October 27^th^ in 2016, using HOBO^®^ temperature loggers (Onset^®^ Comp. Corp.), six times day^-1^, at approx. 0.5m depth (see fig. S1). During this period, temperatures spanned from 1.6 to 26.3 °C. Daily average temperature, during this period, was on average 10.1 °C, max. 20.5 °C and min. 1.9 °C. The within-day range of temperatures (daily max – daily min) was on average 2.6 °C and a minority of the days had profound daily temperature fluctuations of more than 10 °C (5.3% of the sampled days; max. daily temperature range 18.6 °C).


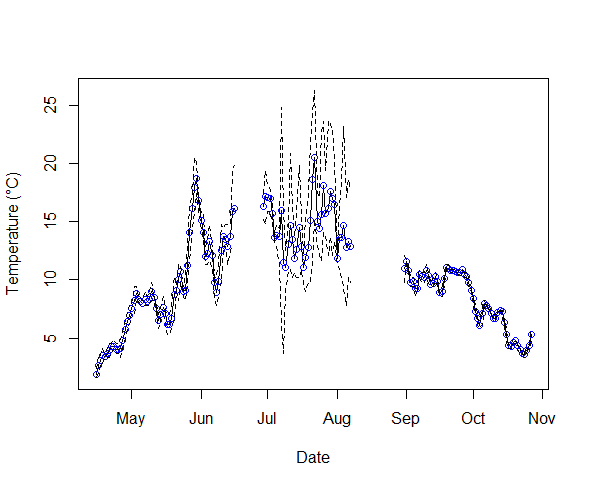


Figure S1 Daily mean water temperatures (blue circles, solid line) at Sandtjønna during the growth season (May – Nov., data from 2016). Dashed lines indicate maximum and minimum daily temperatures (6 measurements day^-1^). During two time intervals (in June & August), some data were excluded due to technical issues with the temperature loggers.


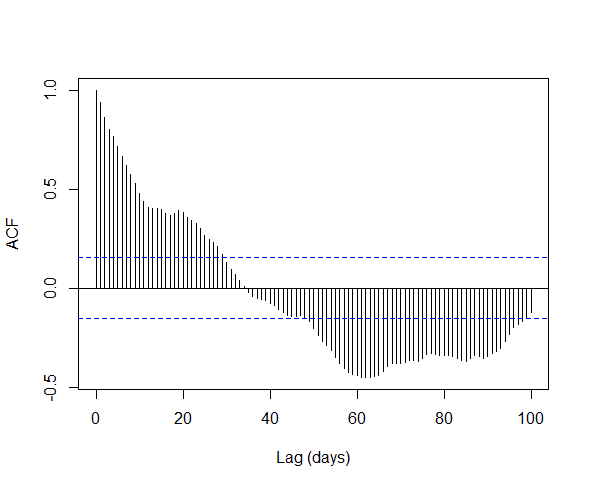


Figure S2 Lag-plot, or correlogram, showing autocorrelation in temperature at Sandtjønna, 2016. The lags represent the number of days (1 lag = 1 day) prior to a specific day (day 0), and the corresponding autocorrelation function (acf) value (Legendre & Legendre, 2012). The generation time for Daphnia magna at 10 °C (mean temperature May - Nov.) is approximately 32 days (Gillooly, 2000).

# References

Gillooly, J.F. 2000. Effect of body size and temperature on generation time in zooplankton. *J. Plankton Res.* **22**: 241-251.

Legendre, P. & Legendre, L. 2012. Chapter 12 - Ecological data series. In: *Developments in Environmental Modelling* Vol. 24 (Pierre, L. & Louis, L., eds)*.* pp. 711-783. Elsevier, Amsterdam, Boston.
